# Chitosan-2D Nanomaterial-Based Scaffolds for Biomedical Applications

**DOI:** 10.3390/polym16101327

**Published:** 2024-05-08

**Authors:** Atanu Naskar, Sreenivasulu Kilari, Sanjay Misra

**Affiliations:** Vascular and Interventional Radiology Translational Laboratory, Division of Vascular and Interventional Radiology, Department of Radiology, Mayo Clinic, Rochester, MN 55905, USA; naskar.atanu@mayo.edu (A.N.); kilari.sreenivasulu@mayo.edu (S.K.)

**Keywords:** chitosan, 2D nanomaterials, scaffold, biomedical applications, biomaterials

## Abstract

Chitosan (CS) and two-dimensional nanomaterial (2D nanomaterials)-based scaffolds have received widespread attention in recent times in biomedical applications due to their excellent synergistic potential. CS has garnered much attention as a biomedical scaffold material either alone or in combination with some other material due to its favorable physiochemical properties. The emerging 2D nanomaterials, such as black phosphorus (BP), molybdenum disulfide (MoS_2_), etc., have taken huge steps towards varying biomedical applications. However, the implementation of a CS-2D nanomaterial-based scaffold for clinical applications remains challenging for different reasons such as toxicity, stability, etc. Here, we reviewed different types of CS scaffold materials and discussed their advantages in biomedical applications. In addition, a different CS nanostructure, instead of a scaffold, has been described. After that, the importance of 2D nanomaterials has been elaborated on in terms of physiochemical properties. In the next section, the biomedical applications of CS with different 2D nanomaterial scaffolds have been highlighted. Finally, we highlighted the existing challenges and future perspectives of using CS-2D nanomaterial scaffolds for biomedical applications. We hope that this review will encourage a more synergistic biomedical application of the CS-2D nanomaterial scaffolds and their utilization clinical applications.

## 1. Introduction

Chitosan (CS) is a cationic natural linear polysaccharide of β-(1→4)-linked D-glucosamine and N-acetyl-D-glucosamine derived from the alkaline hydrolysis of chitin. CS is the second most naturally abundant biopolymer after cellulose and is one of the most vastly used natural materials in the medicine, agriculture, and food processing industries due its biocompatibility, biodegradability, and physiochemical properties [1,2]. Therefore, CS has been classified as a generally recognized as safe (GRAS) compound by the Food and Drug Administration (FDA) [3]. This is why numerous researchers have extensively followed CS or CS-based scaffolds for biomedical applications, which can be confirmed by the abundant published work based on CS [4,5,6].

Because of the structural similarity of CS to glycosaminoglycans, as one of the components in extracellular matrix (ECM) [7], CS scaffolds are used in biomedical research and the development of therapeutics. Due to its structural advantages, CS scaffolds are also utilized for various vascular regeneration applications [8,9]. Although CS preparation is cost-effective, poor solubility and porosity are limiting factors for their usage in a wide range of biomedical applications [10,11]. However, CS can combine with other polymers, metal, metal oxide, and 2D nanomaterials to augment those properties and lead to scaffold formation [12]. In this respect, 2D nanomaterials, such as graphene, black phosphorus, and MoS_2_, when combined with CS scaffold have shown synergistic properties [13,14,15]. Further, due to the large surface area of 2D nanomaterials, the chance of cell interaction with 2D materials is higher. However, the nanomaterials have their own toxicity issue which can be overcome by CS capping [16]. Hence, not only can 2D nanomaterials be utilized with CS, but it can be synergistically applied for nanomaterial applications.

Here, we discuss the advantages of CS biomaterial scaffolds and the functionalized synergistic applications of the CS scaffolds in combination with 2D materials. Finally, the prospects and challenges of CS-based 2D materials scaffold in clinical applications are discussed.

## 2. Advantages of Chitosan for Biomedical Applications

The biomedical applications of a biomaterial are dependent on their favorable physiochemical properties such as porosity, solubility, biodegradability, biocompatibility, etc. In this section, we discuss the chitosan properties that make CS an excellent scaffold biomaterial in biomedical applications.

### 2.1. Biocompatibility

The biocompatibility of a material can be determined by its compatibility with the biological system with minimal or no adverse effects, including immunogenicity in vivo. The CS materials are relatively non-toxic excellent biocompatible materials which are derivatives of chitin. A highly conserved extracellular matrix appeared across the animals from invertebrates to higher mammals. Moreover, upon degradation, CS releases its constitutive ingredients, D-glucosamine and N-acetyl-D-glucosamine, which are natural components that can be utilized for tissue regeneration and in the healing process. [10]. Although, CS has been extensively investigated as a nanobiomaterial due to its non-toxicity, biodegradability, and biocompatibility and granted FDA Generally Recognized As Safe (GRAS) status (GRN n° 73, 170, 397 and 443), some studies showed toxic effects after using CS in the cell lines of zebrafish [17,18].

### 2.2. Porosity

Porosity is one of the important features for any scaffold to determine its biocompatibility. Cell adhesion to the scaffold material is dependent on the pore size of the scaffold, which, if too small, results limited cell permeability, whereas too large pores result in a limited surface area and reduced ligand density for the cell to bind. Therefore, it is important to maintain optimal pore size for cell adhesion and growth [19]. Further, different pore size scaffolds are required for certain types of applications in biomedical research. For example, a scaffold with greater than 20–100 µm is a good fit for cell infiltration [20], while more than 100 µm is well recommended for neovascularization studies [21]. Similarly, a scaffold up to 300 µm pore size is ideal for endochondral ossification, whereas pores above 300 µm in scaffolds showed in osteogenesis studies [22]. Therefore, it is necessary to select the appropriate pore size for specific applications.

The pore size of the scaffold can be controlled by regulating the temperature and water content in the scaffolds. For example, the lower the temperature, the greater the water content and the smaller the pore size. Further, the thermal-induced phase separation (TIPS) method is used to synthesize different structures with different pores [23].

Further, the pore size of the CS scaffold also depends on various parameters, such as crosslinkers, freezing temperature, the concentration of polymer, and the addition of other compounds such as drug, nanoparticles, etc. For example, Shavandi et al. [24] demonstrated that the CS scaffold with hydroxyapatite and beta-tricalcium phosphate, prepared at −80 °C and −20 °C vary in their pore size. The CS scaffolds that were prepared at −80 °C, showed elongated pores with an irregularity in shape, whereas the scaffold of −20 °C showed highly layered pores with more irregularities. Similarly, the addition of hydroxyapatite nanoparticles to CS-silk fibroin (SF) scaffold has a reduced porosity compared to the CS-silk fibroin (SF) scaffold [25].

### 2.3. Molecular Weight

CS is a polysaccharide of D-glucosamine and N-acetyl-D-glucosamine units, and the molecular weight varies with the number of D-glucosamine and N-acetyl-D-glucosamine units. The physiochemical and biological properties, including solubility and the viscosity of CS changes with increases in the molecular weight [26]. Depending on the source and preparation process, the molecular weight of CS ranges from ~300 to 1000 kDa [27]. CS with a higher molecular weight becomes more viscous, less soluble, and consequently less permeable, which is not desirable for various biomedical applications. Hence, low molecular weight CS is commonly used in CS scaffold preparation for biomedical applications, due to its excellent solubility and stability [28].

### 2.4. Water Retention Ability

The water retention ability of any scaffold material can be described as the ability to swell and hold certain volumes of water after being placed in a liquid medium [29]. The scaffolds materials water absorption resulted in increased pore size and swelling. The content of aminosugars in CS determine its swelling ability [30]. The cationic CS materials have an electrostatic interaction with anionic polymers, resulting in polymeric complexation and decreased swelling. In this context, the inclusion of silicon dioxide and zirconia nano particles significantly reduced the swelling behavior of the CS scaffold [31]. The addition of bioactive glass ceramic nanoparticles (nBGC) to the CS–gelatin scaffold have shown a significant reduction in the swelling ability of the CS scaffold [32]. Together, these reports indicate that the swelling ability of CS scaffolds can be modified as needed.

### 2.5. Biodegradability

The process of degradation and he longevity of a scaffold material in the biological system are key factors in selecting the biomaterial therapeutics [33]. The process of degradation can be hydrolysis and or enzymatic, and the resulting degraded products should be non-immunogenic and non-toxic and are incorporated into metabolic pathways or excreted [34]. As previously mentioned, the CS materials are derived from the chitin one of the extracellular components in the biological systems, which is highly conserved across the species and, therefore, CS materials are non-toxic and minimal immunogenic. The CS is hydrolyzed to acetylated and amino sugars which may be re-cycled or excreted [35]. The rate of CS degradation also depends on degree of deacetylation and hydrolysis by lysozyme [36]. However, due to its high degradation rates, the usage of CS scaffolds in vivo for long term application is limited. The addition of nanoparticles or other polymers into the CS scaffold seemed to affect the degradation rate. For example, Saravanan et al. [37] showed that the addition of nano-hydroxyapatite (nHAp) into the CS scaffold seemed to accelerate the derogation rate, while the opposite result can be seen after the addition of nano silver (nAg) in the CS matrix. Similarly, the addition of bioactive glass ceramic nanoparticles (nBGC) to the CS–gelatin scaffold considerably reduced their degradation rate [32].

## 3. Types of Chitosan Scaffold

CS materials, such as hydrogel, sponges nanofiber membrane, etc., have been used in various biomedical applications, including wound healing and tissue engineering. In this section, we discuss the different types of CS scaffold used in tissue engineering.

### 3.1. Hydrogel Scaffold

Hydrogels are cross-linked and a polymeric network of hydrophilic units and the gelation can be initiated via physical and or chemical reactions [38]. Hydrogel-based scaffolds are supporting materials that have the potential to mimic the extracellular matrix, which provides cell–cell communications with the sustained release of water and other biomolecules for tissue regeneration and the healing process [39]. The hydrophilic structure of the hydrogel scaffold gives it the capability to maintain considerable amounts of water or other biological fluids, which helps in nutrient diffusion. It is worthy to note that a proper hydrogel should be able to regenerate specific tissues, while achieving the minimum requirements for vascularization, cell growth, proliferation, and concurrent degradation during the healing process, along with its biocompatible and non-toxic properties [40]. Superior physical and mechanical stability, high biodegradability, and high durability are some of the other characteristics of a proper hydrogel scaffold. The advantages of a CS hydrogel scaffold are its excellent inherent biodegradability, biocompatibility, and hydrophilic surface. However, its extreme viscosity, combined with its mechanical weakness, are some of the limitations which are yet to be resolved [40].

In recent years, the urgency to develop smart injectable hydrogels has increased due to its minimal invasive approach. Smart injectable hydrogels are liquid at room temperature but form a gel when injected into a fractured location, which has the potential for scar size reduction, less post-operative pain, the rapid recovery of patients, and obvious cost-effectiveness [41]. Naturally occurring polysaccharides are especially relevant to hydrogel preparation as they mimics many features of the extracellular matrix. Chitosan, a naturally occurring polysaccharide and a pH-responsive polymer is significant in this scenario [42]. The anionic nature of most human tissues can perfectly adhere to the cationic character of chitosan and the subsequent adherence of CS hydrogels to tissue sites [43]. Additionally, the polycationic nature of chitosan enabled the preparation of cross-linked hydrogels without any use of cross-linking agents, which might be toxic.

### 3.2. Sponges

The primary advantage of chitosan sponges is that its micro-porous structure enables it to absorb high amounts of fluids. In some cases, this amount of absorbed fluid is 20 times more than its dry weight, without compromising its flexibility and texture [44]. With respect to wound healing applications, CS sponges prevent contamination in wound and dehydration due to its porous structure [45]. For example, CS/tricalcium phosphate [46], CS/collagen sponges [47] are used as scaffolds in bone regeneration. Du et al. [48] showed the excellent wound healing potential of micro-channeled alkylated chitosan sponge, which are able to guide in situ tissue regeneration for noncompressible hemorrhages. In another example, Wu et al. [49] prepared ampicillin-grafted chitosan sponges as an antibacterial material against *Staphylococcus aureus*, *Candida albicans*, and *Escherichia coli* and showed its potential as a wound dressing material. In a similar experiment, Al-Mofty et al. [50] showed the antibacterial and hemostatic activity of PVA/chitosan sponges loaded with hydroxyapatite and ciprofloxacin. It is worthy to note that CS sponges also have some disadvantages such as poor mechanical properties and rapid degradation prepared in acidic conditions, which hindered its growth in application processes [51].

### 3.3. Fiber Scaffolds

Fiber scaffolds were generally utilized to disperse the bioactive agents within the fibrous matrix. The bioactive agents either can also be adsorbed on the surface of the fibers or blended into the electrospinning polymer solution to produce fiber scaffolds [52]. The release of bioactive molecules from the fiber scaffold is straightforward where the fiber scaffolds usually burst release due to the dissolution of bioactive agents [53]. However, despite the simplicity of the process, the release rate of the bioactive agents directly depends on the degradation rate of the polymer matrix. Moreover, the solvents in the electrospinning solution utilized to disperse the bioactive agents can also hinder the activity of the molecules. Fiber-based chitosan scaffolds were also utilized to resolve the high viscosity problem of chitosan [54]. In this case, a nanofiber diameter of 140 nm can be achieved with chitosan that is hydrolyzed for 48 h. Additionally, electrospinning conditions and the solvent concentration also affected the fiber diameter.

### 3.4. Microspheres Scaffolds

CS microsphere scaffolds have been used for controlled drug release and increased bioavailability [55]. The preparation of the CS microsphere was enabled after reacting chitosan with controlled amounts of multivalent anion, which, in turn, resulted in cross-linking between the chitosan molecules [55]. Precipitation, cross-linking with anions, modified emulsification, thermal cross-linking, etc., are some the techniques utilized to prepare the CS microsphere [56]. The nature of drug molecule which needs to be incorporated into CS microsphere decides the selection of preparation process.

Hu et al. [57] utilized a combination of biodegradable poly-(lactic acid-co-trimethylene carbonate) and chitosan microspheres for bone tissue engineering. The porosity, pore size, and mechanical properties of these CS microsphere scaffolds can be controlled through the preparation methods and parameters. Moreover, this CS microsphere-based scaffolds possessed shape-memory effects, i.e., it can recover to its initial shape when heated to 37 °C within 300 s. The scaffold has the potential for bone regeneration applications. In another example, Budhiraja et al. [58] exploited the formulation of mupirocin-loaded chitosan microspheres embedded in Piper betle extract containing a collagen scaffold for the purpose of wound healing activity. Similarly, Fan et al. [59] showed the effectiveness of covalent and injectable chitosan-chondroitin sulfate hydrogels embedded with chitosan microspheres as an injectable drug and cell delivery system in cartilage tissue engineering. The porosity, pore size, and mechanical properties of these CS microsphere scaffolds can be tuned through the preparation methods. Moreover, these CS microsphere scaffolds regain their shape upon heating to 37 °C within 300 s. Such a shape memory effect is favorable for spatial implantation applications.

## 4. Types of Chitosan Nanostructures

Chitosan is used as hydrogel scaffolding material [8]. However, the poor mechanical properties of CS hydrogel or CS films have hindered their application in scaffolds, despite their having excellent biomedical properties [60]. Hence, efforts have been made to incorporate different forms of CS, such as CS nanoparticles, CS nanosphere, CS nanosheets.

### 4.1. Chitosan Nanoparticles (CS NPs)

Chitosan NPs have been successfully utilized because of their mucoadhesive capacity, enhanced bioavailability, non-toxic, and biocompatibility, etc. [61]. Additionally, CS NPs have a large surface-to-volume ratio which, in turn, enables it to provide a great binding capacity for biological macromolecules in various biomedical applications [62]. Moreover, the growth factors and signaling molecules can be easily loaded into the scaffolding materials through the incorporation of CS NPs [63].

Further, the addition of CS NPs to the scaffolding materials have showed an enhanced biocompatibility and accelerated hydrolytic degradation for potential in tissue engineering applications as listed in Table 1.

### 4.2. Chitosan Nanospheres (CS NSs)

Chitosan nanospheres (CS NSs) is another nanomaterial which is used for drug delivery application mainly because of its high surface area, excellent porosity, effective chemical stability, and stable geometric structure [71,72,73].

There are various examples of CS NSs as a nanocomposite material or as a scaffold material in biomedical applications. For example, Yang et al. [71] synthesized an injectable carboxymethyl chitosan/nanosphere-based hydrogel for drug release and lubrication in ameliorating from arthritis. The average size of the NP utilized in this hydrogel was in the range of 47.7 nm to 52.1 nm. Moreover, CS NSs are also used for the delivery of the anticancer drug 5-fluorouracil [72]. The mean diameter of CS NSs was ~200 nm. However, despite its potential for excellent biomedical applications, its particle size and morphology are not fully controllable, which limits its potential in biomedical applications.

### 4.3. Chitosan Nanosheets (CS NTs)

Chitosan nanosheets are another nanostructure which have shown excellent potential for biomedical applications. There are limited studies using CS NTs in wound healing activities with lower inflammatory cells infiltration, along with new epithelium thickness [74].

## 5. The Advantages of 2D Nanomaterials for Biomedical Applications

Two-dimensional nanomaterials such as graphene, black phosphorus, metal carbides, and nitrides (MXenes), etc., have shown excellent potential as biomaterials in various biomedical applications [75,76]. Recent studies on the utilization of 2D nanomaterials in biomedical research can be attributed to their excellent physiochemical properties, [77,78] which makes them attractive candidates for biosensing, bioimaging, drug delivery, and regenerative medicine. Another advantage of various 2D nanomaterials is that they can be utilized with CS or some other polymer material for synergistic biomedical applications, i.e., the 2D material-based CS nanocomposite would show much improved biomedical properties than individual samples. There are several advantages of 2D nano materials such as:

- High surface-to-volume ratio and tunable interfacial chemistry are some of the most important characteristics of 2D nanomaterials, which are generally required for biomedical applications.
- 2D nanomaterials showed a rippling or wrinkling effect in the case of out-of-plane bending or folding, which allows cells to strongly attach and spread freely over the underlying substrate [79]. This process of nanocomposite formation helped in biomedical applications as strong cell attachment to the substrate is one of the desired criteria for biomedical applications.
- Mechanical strain gradients allow electrical polarization, which can regenerate electrically active tissues such as bone, neurons, and cardiac tissue [80].
- Two-dimensional nanomaterials can interact with cellular membrane in penetration mode as well as attachment mode [79,81]. Hydrophobic attraction drives the penetration mode interaction between the lipid layer of cellular membrane and the 2D nanomaterials, whereas the hydrophilic interaction works for the interaction in attachment mode.
- The lateral size of the 2D nanomaterials also determine the interaction mode between the cellular membrane and 2D nanomaterials [79,81]. For example, nanomaterials with similar dimensions to plasma membrane implement attachment mode, whereas larger dimension nanomaterials utilize penetration mode.

## 6. Chitosan-2D Nanomaterial Scaffolds for Biomedical Applications

In recent years, 2D nanomaterials such as graphene, black phosphorus (BP), MoS_2_, were increasingly utilized for various biomedical applications. A combination of chitosan with 2D nanomaterials used for synergistic biomedical applications are listed in Table 2.

### 6.1. Chitosan-Graphene

Graphene is a derivative of graphite, a thin 2D nanomaterial with high tensile strength and electrical conductivity. Although there are promising results, the biocompatibility of graphene is under debate. The addition of graphene 2D material to CS scaffold has shown synergistic effects, tissue regeneration, and cardiac repair [13,84]. Hermenean et al. [82] exploited CS-graphene oxide (GO) 3D scaffolds for bone tissue regeneration in critical-size mouse calvarial defects. When combined with GO, CS scaffolds showed the synergistic increment of alkaline phosphatase activity both in vitro and in vivo experiments, along with an increased expression of bone morphogenetic protein (BMP) and Runx-2, and showed its bone tissue regeneration ability. In a similar approach, Dinescu et al. [83] used the GO with a CS-based 3D scaffold, which showed the formation of ordered morphologies and a higher total porosity, combined with a greater surface availability for cell attachment.

**Figure 1:**
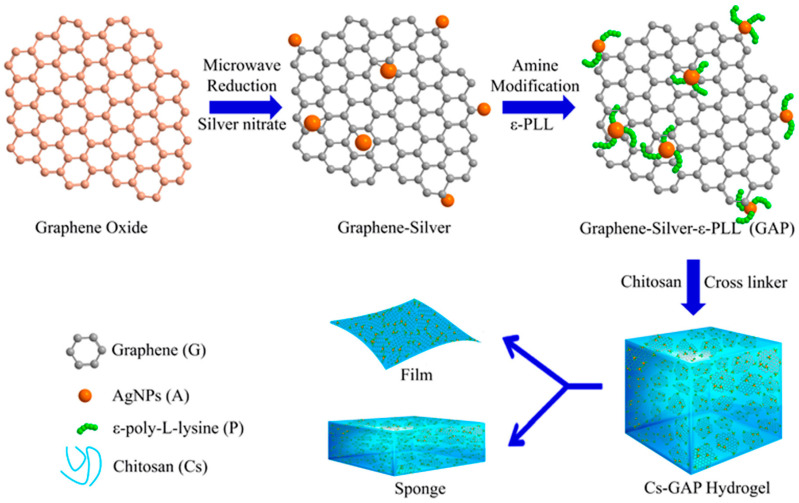
Schematic representation for the preparation of CS-GAP nanobiocomposite film and sponge. Reproduced with permission from Ref. [13] Copyright 2020, American Chemical Society.

CS-based graphene nanocomposites were successfully experimented on as antibacterial scaffolds in hemorrhage control and wound-healing applications [13]. The nanobiocomposite scaffolds were fabricated by the incorporation of graphene-silver-polycationic peptide (GAP) nanocomposite into CS (Figure 1). One of the CS-GAP scaffolds showed excellent antibacterial activity against *E. coli* and *S. aureus*, along with excellent porosity, fluid absorption, and mechanical strength. Saravanan et al. [84] also showed the importance of GO and Au nanosheet-based CS scaffolds for the improvement of ventricular contractility and function into Infarcted Heart. The particle size of Au NPs was ~8 nm utilized in the nanocomposite. In another experiment, Sivashankari et al. [85] exploited agarose/CS/graphene composite scaffolds for its potential application in bone and osteochondral tissue engineering. The functional recovery of injured spinal cord in rats was successfully achieved through using GO composite-based CS scaffolds [86]. The various examples of cartilage tissue engineering [87] and cardiac tissue engineering [88] was also achieved through using synergistic GO-based CS scaffolds.

### 6.2. Chitosan-Black Phosphorus

**Figure 2:**
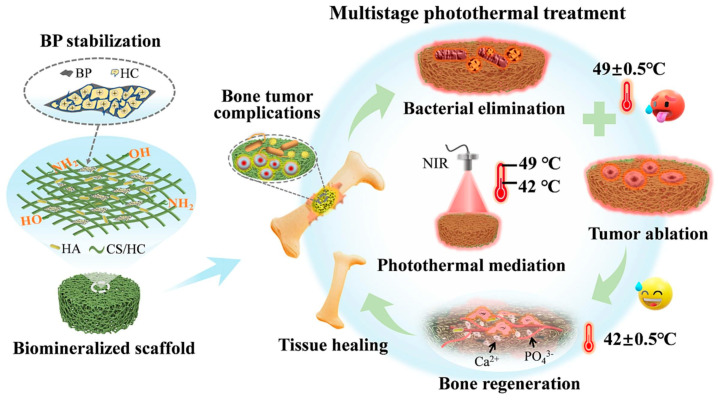
Schematic representation of the Biomineralized CS/HC/HA/BP scaffold formation for the photothermal treatment of bacterial elimination, tumor ablation, and subsequent osteogenesis. Reproduced with permission from Ref. [14] Copyright 2023, Elsevier.

Black phosphorus (BP) has risen up with huge potential due to its favorable physiochemical properties [97,98,99]. Unlike graphene, BP has its own biocompatible and biodegradable properties. It showed excellent photo active properties, as well as photothermal anticancer or antibacterial applications [99,100]. It is worthy to note that the degradation products of BP are safe PO_4_^3−^ and are capable of enhancing the osteogenesis process. However, the instability of BP hindered its usage in biomedical applications [77,99]. Zhao et al. [14] used chitosan/hydroxyapatite/black phosphorus (CS/HC/HA/BP) hybrid photothermal scaffold (Figure 2) to solve bone tumor-related complications. CS not only stabilized the BP-based scaffold but also synergistically act for simultaneous antitumor/antibacterial properties under the photothermal stimulation of <50 °C. In another work, BP nanosheets were combined with platelet-rich plasma (PRP)-chitosan thermo responsive hydrogel for the preparation of a therapeutic platform for the phototherapy treatment of rheumatoid arthritis [89]. This injectable CS thermo-responsive hydrogel was able to control the degradation products of the BP nanosheets, which were simultaneously used as raw materials for osteanagenesis. Moreover, this hydrogel could protect articular cartilage by reducing the friction on the surrounding tissue. Similarly, He et al. [90] prepared layer-by-layer assembled BP/CS composite coating for a multi-functional bone scaffold for osteosarcoma management and bone repair. Th size of BP was ~200 nm. The biocompatible polyetheretherketone (PEEK) scaffold provided similar mechanical properties and architecture compared to that of the natural bone.

### 6.3. Chitosan-MoS_2_

Among the 2D-layered transition metal dichalcogenides, molybdenum disulfide (MoS_2_) in particular, has shown promising results for applications in environmental and biomedical fields [101,102]. In this regard, Mutalik et al. [103] showed the potential of the phase dependent. The MoS_2_-based hydrogels showed excellent mechanical properties, along with intrinsic NIR region absorption for useful photothermal conversion efficiency [104]. However, the negative charge surface of MoS_2_ limits its interaction with cells [91]. Therefore, coating of MoS_2_ with a cationic biocompatible agent such as CS seemed to be an excellent strategy for more cellular interaction with synergistic nanocomposites. Additionally, the poor hydrophilic property of MoS_2_ can be modified with CS coating. Therefore, the combination of CS with MoS_2_ seems to have lots of synergistic potential for various biomedical applications.

**Figure 3:**
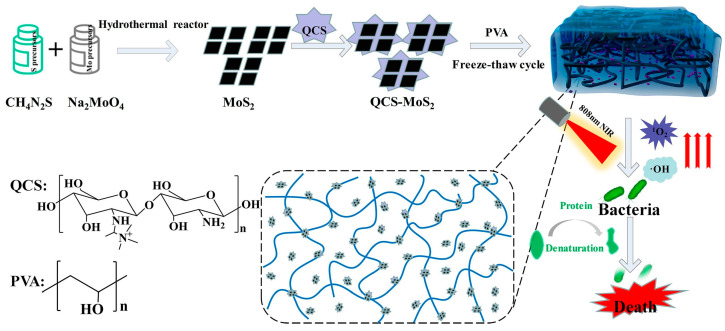
Schematic representation of the QCS-MoS_2_/PVA hydrogel formation for photothermal treatment of bacterial elimination. Reproduced with permission from Ref. [15]. Copyright 2022, Elsevier.

Yan et al. [15] used a quaternized chitosan (QCS)-coated MoS_2_/poly(vinyl alcohol) hydrogel (Figure 3) for NIR-responsive photothermal antibacterial activity against *S. aureus* and *E. coli*. The incorporation of QCS- MoS_2_ seemed to increase the mechanical properties of the hydrogel. Similarly, Shen et al. [91], developed in situ grown bacterial cellulose/MoS_2_-chitosan nanocomposite (BC/MoS_2_-CS) for excellent photodynamic and photothermal antibacterial activities against *E. coli* and *S. aureus* under visible-light illumination. The cationic CS coating enabled the nanocomposite for more bacteria interaction, which eventually led to bacteria cell killing. Moreover, CS also seemed to potentiate the antibacterial activity of the nanocomposite by bacterial membrane disruption and/or permeability.

MoS_2_-based CS hydrogels were also utilized for colon cancer treatment [92]. In this experiment, MoS_2_ nanoflower was doped into CS/oxidized dextran hydrogels and then used for sequential delivery of methotrexate (MTX) and 5-Fluorouracil (5-FU). The NIR irradiation onto the nanocomposite generated hyperthermia due to the presence of MoS_2_, which led to the consequent release of 5-FU encapsulated. In other experiments, Xu et al. [93] and Mukheem et al. [94] showed photothermal antibacterial activity of CS-based MoS_2_ hydrogels.

### 6.4. Chitosan-MXene

Transition metal carbide (MXene) is another 2D material which has shown excellent photothermal properties and biocompatibility, which can be utilized for various biomedical applications [105]. However, it also tends to aggregate in the physiological environment, which limits its usage in biological applications. CS, in combination with MXene, has been shown to be a stable nanocomposite [95,105]. Further, CS and MXene combination shows higher porosity, which is an essential criterion for any functional hydrogel.

Dong et al. [95] prepared Ti_3_C_2_T_x_ MXene-loaded chitosan (MX-CS) hydrogel for photothermal synergistic activity against methicillin-resistant *S. aureus*. The MX-CS hydrogel not only adsorb MRSA cells via CS-MRSA interactions, but it can also kill the bacteria by NIR-irradiated photothermal hyperthermia. In another study, the porosity of CS-hyaluronate matrix hydrogel nanocomposites was controlled by the addition of 2D Ti3C2Tx MXene [11]. Due to the large porosity of the nanocomposite, a small amount of MXene (1–5 wt.%) in CS-based hydrogel was effective against *E. coli*, *S. aureus*, and Bacillus sp. bacteria. In a different application, Liu et al. [96] utilized a MXene@CS based conductive polyacrylamide hydrogel for highly stretchable and sensitive wearable skin.

## 7. Conclusions and Future Perspectives

Chitosan-based materials were utilized for many biomedical applications, such as antibacterial activity, wound healing, anticancer activity, and tissue engineering (bone, cartilage, cardiac, dental, skin, etc.) applications, etc. However, there are certain limitations of CS alone as a scaffolding material in biomedical applications which include: (1) lacking sufficient mechanical strength, (2) porosity, and (3) solubility. However, the current research shows that the combination of CS with other 2D nanomaterials not only overcomes these limitations but also seems to complement and elicit synergistic effects, including the mechanical strength and porosity. In short, it is valid to say that significant progress has been made for CS-based scaffolds and their biomedical applications. Two-dimensional nanomaterial-based CS scaffolds also showed great promise in clinical application. Additionally, CS-2D nanomaterials scaffolds can also be experimented for wrapping around the vessels in vascular surgery procedures, as previously performed through CorMatrix in our lab [106]. However, significant challenges, such as the ineffective delivery of growth and large scale reproducibility, still need to be overcome as the translation from the lab scale into clinical trials has been limited due to the industrial-scale production quantities and quality. Hence, a different approach for large scale production, such as cross flow filtration (CFF) [107], or some other process to enhance the large-scale production of CS should be executed. Overall, it is fair to conclude that, despite having some limitations to deal with, CS is an excellent base material, whereas other nanomaterials, such as 2D nanomaterials, can be utilized for more direction- and application-oriented research work.

## Figures and Tables

**Table 1:** Chitosan nanoparticle (CS NP)-based scaffolds and their applications.

Material	Effect	NP Size	Ref.
CS NPs-BSA-bFGF	Significantly affected the physical properties of chitosan-gelatin scaffold	∼266 nm	[64]
CUR-CS NPs	Improved stability and solubility for better tissue regeneration applications	∼197 nm	[65]
GelMA/CS NPs-bFGF	Provide a sustained release of growth factors	∼267 nm	[66]
CS NPs-PCL-DEX	Enhanced osteogenic differentiation of the mesenchymal stem cells	∼285 nm	[67]
PVA NF with SIM/CS NPs	Controlled drug delivery for bone regeneration application	∼110–140 nm	[68]
GA-CSNPs	Wound healing	∼96–357 nm	[69]
CS NPs-PHB	Cartilage tissue engineering	∼255 nm	[70]

**Abbreviations:** CS NPs: Chitosan nanoparticles, BSA: bovine serum albumin, CUR: curcumin, GelMA: Gelatin methacryloyl, PCL: poly-ε-caprolacton, DEX: dexamethasone, PVA NF: Polyvinyl alcohol nanofiber, SIM: Simvastatin, GA: Gallic acid, PHB: polyhydroxy butyrate.

**Table 2:** Chitosan (CS)-2D nanomaterial-based scaffolds and their applications.

Material	Effect	Ref.
CS-GO-1	Bone tissue regeneration in critical-size mouse calvarial defects	[82]
CS-GO-2	Ability to support stem cell differentiation processes for bone tissue engineering	[83]
CS-GAP	Antibacterial scaffolds for hemorrhage control and wound-healing application	[13]
CS-GO-Au	Improvement of the ventricular contractility and function into infarcted heart in rat model.	[84]
Agarose/CS/GO	Potential application in bone and osteochondral tissue engineering	[85]
GO-composited CS	Functional recovery of injured spinal cord in rats	[86]
CS-GO-3	Cartilage tissue engineering	[87]
GO/CS	Cardiac tissue engineering	[88]
CS/HC/HA/BP	Photothermal scaffold for bone tumor-related application	[14]
BP/CS/PRP	Photothermal treatment of rheumatoid arthritis	[89]
BP/CS composite	The biocompatible polyetheretherketone (PEEK) scaffold provided similar mechanical properties and architecture compared to that of the natural bone.	[90]
QCS-MoS_2_-PVA	Photothermal antibacterial activity against *S. aureus* and *E. coli*.	[15]
BC/MoS_2_-CS	Photodynamic and photothermal antibacterial activities against *E. coli* and *S. aureus*	[91]
MoS_2_ doped CS/OD hydrogels	Photothermal colon cancer treatment	[92]
MoS_2_-LA-COS	Photothermal antibacterial activity against *S. aureus* and *E. coli*.	[93]
PHA-CS/MoS_2_	Antibacterial activity against multi-drug-resistant *E. coli* K1 and methicillin-resistant *S. aureus* (MRSA)	[94]
MX-CS	Synergistic photothermal antibacterial activity against MRSA	[95]
MX-CS-hyaluronate	Antibacterial activity against *E. coli*, *S. aureus*, and *Bacillus* sp.	[11]
MXene@CS	Highly stretchable and sensitive wearable skin	[96]

**Abbreviations:** CS: Chitosan, GO: Graphene oxide, GAP: graphene-silver-polycationic peptide, HC: hydroxypropyltrimethyl ammonium chloride chitosan, HA: hydroxyapatite, BP: black phosphorus, PRP: platelet-rich plasma, QCS: quaternized chitosan, BC: Bacterial cellulose, OD: oxidized dextran, LA: α-lipoic acid, COS: chitosan oligosaccharide, PHA: polyhydroxyalkanoate, MX: Ti_3_C_2_T_x_ MXene.

## Data Availability

Not applicable.
